# Metabolic Dysfunction‐Associated Steatotic Disease and Genetic Risk in Germany—An Observational Real‐World Cohort Study

**DOI:** 10.1002/ueg2.70298

**Published:** 2026-10-07

**Authors:** Marcin Krawczyk, Frank Grünhage, Lisa Schubert, Nektarios Dikopoulos, Jeannette Schwenzer, Marion Muche, Johannes Wiegand, Gisela Felten, Renate Heyne, Patrick Ingiliz, Anna Schmidt, Kerstin Stein, Susanne N. Weber, Heiner Wedemeyer, Stefan Zeuzem, Peter Buggisch, Frank Lammert, Andreas Geier, Wolf Peter Hofmann, Jörn M. Schattenberg

**Affiliations:** ^1^ Department of Gastroenterology, Hepatology and Transplant Medicine Medical Faculty University of Duisburg‐Essen Essen Germany; ^2^ KRH Klinikum Siloah Hannover Germany; ^3^ Gastroenterologie am Bayerischen Platz Berlin Germany; ^4^ Gastroenterologische Schwerpunktpraxis Dornstadt Germany; ^5^ Bauchzentrum Biesdorf Berlin Germany; ^6^ Charité Campus Benjamin Franklin Berlin Germany; ^7^ Division of Hepatology Department of Medicine II Leipzig University Medical Center Leipzig Germany; ^8^ Gastroenterologische Praxis Herne Germany; ^9^ Leberzentrum am Checkpoint Berlin Germany; ^10^ APHP Hôpital Henri‐Mondor Hepatology Department Inserm U955 Créteil France; ^11^ Magen‐Darm‐Zentrum Wiener Platz Köln Germany; ^12^ Praxis für Infektiologie und Hepatologie Magdeburg Magdeburg‐Altstadt Germany; ^13^ Department of Internal Medicine 2 Saarland University Hospital Homburg Germany; ^14^ Department of Gastroenterology, Hepatology and Endocrinology Hannover Medical School Hannover Germany; ^15^ Department of Internal Medicine I Hospital of the Goethe University Frankfurt Frankfurt Germany; ^16^ IFI Institut für interdisziplinäre Medizin Hamburg Germany; ^17^ bng, Berufsverband Niedergelassener Gastroenterologen e.V Ulm Germany; ^18^ Center for Health Economics Research Hannover (CHERH) Hannover Medical School (MHH) Hannover Germany; ^19^ Division of Hepatology Department of Medicine II University Hospital Würzburg Würzburg Germany; ^20^ Saarland University Saarbrücken Germany

## Abstract

**Background:**

Metabolic dysfunction‐associated steatotic disease (MASLD) affects increasing numbers of patients worldwide. The *PNPLA3* p.I148M variant represents a major genetic determinant of MASLD progression. The loss‐of‐function *HSD17B13* polymorphism rs72613567 was reported in turn to be hepatoprotective.

**Patients:**

We analysed blood samples of 507 patients recruited within the Fatty Liver Assessment in Germany (FLAG) study, of whom 476 (255 males, age 56 years) were genotyped. FLAG is a multicenter real‐world MASLD cohort covering secondary and tertiary healthcare levels. The *PNPLA3* p.I148M and the *HSD17B13* rs72613567 polymorphisms were genotyped using allelic discrimination assays. The control cohort comprised 174 individuals.

**Results:**

The *PNPLA3* p.I148M variant was prevalent among FLAG patients (*p* < 0.001 for the departure from Hardy–Weinberg equilibrium) and increased the risk of developing MASLD (common OR = 2.46, *p* = 6.238e‐09). It was associated with increased ALT and AST, lower serum triglyceride concentrations, and independently with liver stiffness ≥ 9.1 kPa after adjustment for BMI and diabetes (OR = 2.57, *p* = 0.01). Carriers of the *HSD17B13* rs72613567 variant had lower AST. *HSD17B13* heterozygotes had lower FIB‐4 (*p* = 0.028) and lower liver stiffness (*p* = 0.034). Among carriers of the *PNPLA3* p.148M allele, the presence of the *HSD17B13* variant was associated with lower ALT (*p* = 0.023) and AST (*p* = 0.041); however, *HSD17B13* did not abrogate the association of *PNPLA3* with liver fibrosis (adjusted *p* = 0.013).

**Discussion:**

By analysing a real‐world MASLD cohort in Germany, we further underscore the role of the *PNPLA3* variant as the central genetic trigger and modulator of MASLD. The *HSD17B13* polymorphism may exert protective effects in MASLD and could attenuate some *PNPLA3*‐associated effects.

## Key Summary

- Summarize the established knowledge on this subject.
  ◦ Metabolic dysfunction‐associated steatotic liver disease (MASLD) is highly prevalent and can progress to advanced fibrosis, cirrhosis and hepatocellular carcinoma.
  ◦ The *PNPLA3* p.I148M variant is the strongest common genetic risk factor for MASLD and has been associated with steatosis, steatohepatitis, fibrosis and hepatocellular carcinoma.
  ◦ The loss‐of‐function *HSD17B13* rs72613567 variant has been reported to exert hepatoprotective effects and may attenuate liver injury in chronic liver diseases, including MASLD.
  ◦ Limited genetic data for MASLD from real‐world secondary‐ and tertiary care populations in Germany exist.
- What are the significant and/or new findings of this study?
  ◦ In the German multicentre real‐world *FLAG* cohort, the *PNPLA3* p.I148M variant was highly prevalent and was strongly associated with MASLD susceptibility (OR 2.46), elevated ALT and AST levels, and lower serum triglycerides.
  ◦ Homozygosity for the *PNPLA3* risk allele independently increased the risk of significant liver fibrosis (liver stiffness ≥ 9.1 kPa) after adjustment for BMI and type 2 diabetes.
  ◦ Carriers of the *HSD17B13* rs72613567 variant showed lower AST levels and evidence of milder liver disease, including lower FIB‐4 and liver stiffness measurements.
  ◦ Although *HSD17B13* attenuated liver enzyme elevations among *PNPLA3* risk allele carriers, it did not abolish the association between *PNPLA3* and liver fibrosis, confirming *PNPLA3* as the dominant genetic modifier of MASLD severity.

## Graphical abstract

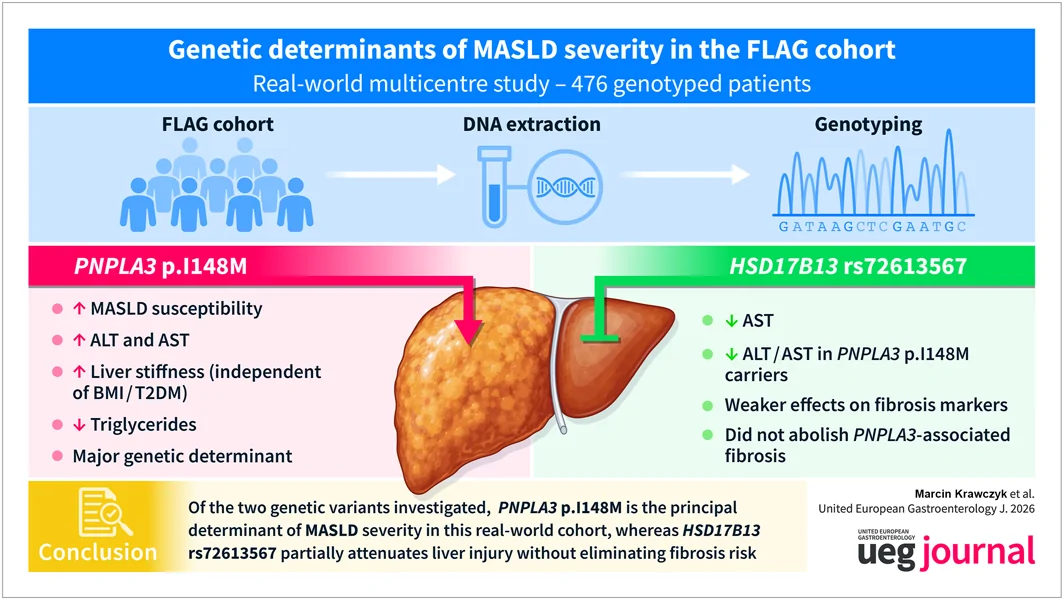

## 1 Introduction

Metabolic dysfunction‐associated steatotic liver disease (MASLD) is one of the most frequent liver conditions with a global prevalence of over 35% [1]. A substantial proportion of individuals with fatty liver develop metabolic‐dysfunction associated steatohepatitis (MASH), which is associated with increased all‐cause morbidity and mortality as well as liver‐specific morbidity potentially leading to cirrhosis and its sequelae including end‐stage liver disease, hepatocellular carcinoma (HCC) and death [2, 3]. The variability in the progression of fatty liver is multifactorial and driven by exogenous as well as inherited determinants. More recently, the development of novel therapies—including genetic aspects of the disease—has brought innovation to patients [4, 5].

MASLD may be suspected in individuals with metabolic syndrome, arterial hypertension, type‐2 diabetes mellitus (T2DM) and hyperlipoproteinemia, as well as, in those who are overweight and have a sedentary lifestyle [6]. The diagnosis of MASLD is based on the presence of hepatic steatosis in the context of metabolic dysfunction and requires assessment of alcohol consumption [6]. Abdominal ultrasound may be indicative of the presence of the fatty liver. A large number of non‐invasive tests to assess the amount of steatosis and to stage liver fibrosis are currently used [7, 8]; nevertheless, the definite diagnosis of MASH still requires liver histology assessment. However, acceptability to liver biopsy is low in both patients and physicians due to the potential risks and inconvenience of this invasive procedure.

In the recent years, numerous genetic risk factors including *PNPLA3, TM6SF2, GCKR, MBOAT7,* and *HSD17B13* have been associated with an increased risk for MASLD, MASH, and HCC [9, 10] In particular, the common variant p.I148M in the *PNPLA3* gene (also known as adiponutrin) was linked to a significantly increased MASLD risk [11, 12]. Following the MRI‐based GWAS study by Romeo et al., which showed that carriers of the minor *PNPLA3* allele are characterised by increased intrahepatic fat contents, it was also linked to an enhanced risk of liver fibrosis, cirrhosis and HCC in patients with metabolic, alcoholic and viral liver diseases [9]. *PNPLA3* is expressed in hepatocytes on the surface of lipid droplets where it possesses the activity of triglyceride lipase [13]. Depending on the analysed population, the *PNPLA3* p.I148M minor allele is present in as much as 50% of individuals [14] and a meta‐analysis [15] showed that it substantially increases the odds of developing MASLD (OR 1.91, 95%CI 1.64–2.21) and MASH (OR 2.54, 2.03–3.16). According to a study by Abul‐Husn [16], genetic variants have not only deleterious effects on fatty liver diseases but might also lower the harmful effects of each other. HSD17B13 is an enzyme that is highly expressed in the liver and is involved in the metabolism of steroids and retinol [17]. In this respect, the *HSD17B13* polymorphism has been shown to have protective effects on the development of MASLD and hepatocellular carcinoma (HCC) [16, 18] and can partially abrogate the harmful effects of the *PNPLA3* polymorphism.

Most of the genetic analyses performed in patients with MASLD come from tertiary university centres. In the study presented here, we analysed individuals with MASLD that were recruited within the Fatty Liver Assessment in Germany (FLAG) program—a real‐world observational cohort study covering mostly the secondary healthcare level. We genotyped the *PNPLA3* and *HSD17B13* polymorphisms to investigate their impact on the setting of secondary care.

## 2 Patients and Methods

### 2.1 Patients

The FLAG study is an observational real‐world cohort study initiated by the Association of Gastroenterologists in Private Practice (Berufsverband Niedergelassener Gastroenterologen Deutschlands) in cooperation with academic medical centres and the German Liver Foundation covering secondary and tertiary healthcare levels [19]. Data collection is performed using an electronic case report form, and data quality is verified by plausibility checks and off‐site monitoring. The study protocol was approved by the local ethics committee (Ärztekammer Berlin, Berlin, Germany; 51/16). Written informed consent was obtained from each patient included in the study. The study protocol conforms to the ethical guidelines of the Declaration of Helsinki.

Data were derived from 13 sites across Germany, including secondary care (office‐based practices) (9/13) and tertiary care (academic outpatient clinics) (4/13). Patient baseline data were recorded between May 2017 and October 2019, resulting in a mean recruitment rate of 17 patients per month.

Inclusion criteria consisted of men and women aged ≥ 18 years in whom diagnosis of MASLD was based on:

- hepatic steatosis assessed by ultrasound or pathological controlled attenuation parameter (CAP) measurements with a cut‐off of 278 dB/m defining hepatic steatosis [20];
- availability of clinical, technical, and laboratory data to compute the fibrosis‐4 (FIB‐4) index, the aspartate‐aminotransferase (AST)‐to‐platelet ratio index (APRI) score, and/or the MASLD fibrosis score as well as liver stiffness measurements (LSMs);
- data availability to assess components of the metabolic syndrome including waist circumference, BMI, presence of arterial hypertension, and type 2 diabetes mellitus; and
- alcohol consumption for men < 30 g/day and women < 20 g/day, respectively.

Alcohol intake was evaluated by frequency (never, occasionally, regular) and categorized according to daily consumption cut‐offs. Patients were excluded if they had evidence of chronic liver diseases other than MASLD, such as alcoholic liver disease, chronic HBV and HCV infections, autoimmune or cholestatic liver disease, haemochromatosis, alpha1‐antitrypsin deficiency, or Wilson disease. Patients who had a history of hepatotoxic medication, such as methotrexate, amiodarone, or long‐term NSAIDs, as well as patients with malignant diseases 12 months before study enrolment were also excluded.

Fasted venous blood samples were drawn for routine biochemical analyses, including liver function tests, and DNA genotyping. A routine hepatological laboratory workup was conducted at the respective expert centres according to local standards ruling out competing liver disease including autoantibody assessment and testing for chronic viral disease. The measures reported herein included the liver enzymes aspartate aminotransferase (AST; U/L), alanine aminotransferase (ALT; U/L), and gamma‐glutamyltransferase (GGT; U/L), Bilirubin (mg/dL), Creatinine (mg/dL), INR, Triglycerides (mg/dL), Total cholesterol (mg/dL), LDL cholesterol (mg/dL), HDL cholesterol (mg/dL), Glucose (mg/dL), HbA1c (%), Ferritin (mg/dL), platelets (/10^9^/L). Importantly, labs were assessed in different laboratories as part of routine clinical assessment. Depending on the availability in the participating centre, liver stiffness measurement (LSM) was performed using the Fibroscan device. The Fibrosis‐4 index (FIB‐4) was calculated as described previously by Sterling et al. [21] with cut‐offs of 1.30 for liver fibrosis stages F0‐F2 and 2.67 for stages F3‐F4 [22]. Analysed transient elastography cut‐offs were set as TE ≥ 9.1 kPa and TE ≥ 13.0 kPa. Cut‐offs were choosen to reflect recent findings in the literature and variabiliy of the test in real world cohorts [23, 24]. The control cohort consisted of 174 healthy adult individuals who underwent colonoscopy with normal findings at our institution and served as the reference group for comparison of genotype frequencies in the population.Further description of the FLAG study cohort including patients baseline characteristics and demographics was published previously [19].

### 2.2 DNA Isolation and Genotyping Procedures

Genotyping of the *PNPLA3* (rs738409) and *HSD17B13* (rs72613567) variants was performed in the GastroLabor at the Department of Internal Medicine II of the Saarland University Hospital by a technician blinded to the phenotypes of patients. DNA was extracted from peripheral blood mononuclear cells using the DNeasy Blood & Tissue Kit (Qiagen) and concentrations of DNA were measured using a spectrophotometer (NanoDrop). *PNPLA3* (rs738409) genotyping was carried out using a commercially available assay (C______7241_10, Thermo Fisher Scientific). For *HSD17B13* (rs72613567), a custom assay was developed by Thermo Fisher Scientific based on the supplied flanking sequence. Both variants were genotyped using TaqMan assays as described [25]. The fluorescence data were analysed with allelic discrimination 7500 Software v.2.0.2.

### 2.3 Statistical Analysis

Unless stated otherwise, all statistical analyses were performed with SPSS 26.0 (SPSS, Munich, Germany) or GraphPad Prism 11.0.2 (GraphPad Software Inc., CA, USA). Quantitative data were expressed as medians and ranges. Exact tests were performed to check the consistency of genotype distribution with Hardy‐Weinberg equilibrium (HWE) (http://ihg.gsf.de/cgi‐bin/hw/hwa1.pl). Genotype frequencies were compared in contingency tables. The Kolmogorov–Smirnov normality test was used to examine the distribution of quantitative traits. Differences in markers of liver injury between the *PNPLA3* and *HSD17B13* genotype groups were evaluated using Mann–Whitney *U*‐test or Student's *t*‐test, ANOVA or Kruskal–Wallis test for continuous variables, as appropriate, and the Chi‐squared test for categorical variables. Effects of studied variants on liver fibrosis were analysed in uni‐ and multivariate regression models (linear or logistic, as appropriate) including BMI and the presence of diabetes mellitus type 2 as covariates.

## 3 Results

### 3.1 Study Cohort and the Results of Genotyping

The characteristics of the FLAG cohort are presented in Table 1 and in our previous manuscript [19]. Among 507 recruited patients, 238 were women, and the median age was 53 years (range: 18–81 years). All patients in the FLAG cohort had MASLD and otherwise no other chronic or acute liver diseases. DNA was available in 476 patients (median age 56 years, range 18–81, 255 males) and both polymorphisms—*PNPLA3* and *HSD17B13*—were successfully genotyped in all these patients. Among them, 348 were recruited in secondary care (office‐based practices), whereas 128 were recruited in tertiary care (academic outpatient clinics).

**TABLE 1:** Clinical characteristics of the FLAG cohort.

Variables	Subject characteristics
*N* (male/female)	476 (255/221)
Age (years)	56 (18–81)
BMI (kg/m^2^)	30.1 (20.2–56.7)
T2DM	30.8%
Transient elastography	
Liver stiffness (kPa) (N, median, range)	227, 6.7 kPa, 2.8–68.9 kPa
≥ 9.1 kPa (N)	67
≥ 13.0 kPa (N)	37
Bilirubin (mg/dL)	0.6 (0.1–10.0)
Creatinine (mg/dL)	0.8 (0.5–1.3)
INR	1.0 (0.8–2.4)
AST (U/L)	38 (16–235)
ALT (U/L)	56 (13–399)
GGT (U/L)	75 (14–827)
ALP (U/L)	80 (24–263)
Triglycerides (mg/dL)	159 (53–812)
Total cholesterol (mg/dL)	201 (42–382)
LDL cholesterol (mg/dL)	124 (40–293)
HDL cholesterol (mg/dL)	47 (19–105)
Glucose (mg/dL)	102 (42–311)
HbA1c (%)	5.8 (4.3–11.3)
Ferritin (mg/dL)	191 (12–1849)
Platelets (×10^9^/L)	235 (30–769)
*HSD17B13* genotypes	
[TT]	287 (60.3%)
[T/TA]	164 (34.4%)
[TA/TA]	25 (5.3%)
*PNPLA3* p.I148M genotypes	
[II]	186 (39.1%)
[IM]	186 (39.1%)
[MM]	104 (21.8%)

*Note:* Values are given as medians (ranges), unless stated otherwise.

Abbreviations: ALP, alkaline phosphatase; ALT, alanine aminotransferase; AST, aspartate transaminase; BMI, body mass index; GGT, gamma‐glutamyl transferase; HbA1c, haemoglobin A1c; HSD17B13, hydroxysteroid 17‐beta dehydrogenase 13; I, isoleucine; INR, International Normalized Ratio; kPa, kilopascal; M, methionine; *PNPLA3*, adiponutrin; T2DM, type 2 diabetes mellitus.

### 3.2 High *PNPLA3* Allelic Frequencies in Secondary Care and Associations With Clinical Diagnosis of MASLD

The *PNPLA3* p.I148M variant was overrepresented in the FLAG cohort: the allele [M] was detected in 60.9% of recruited patients, which led to a significant deterioration of the entire cohort from the HWE (*p* < 0.001 according to the exact test, Supporting Information S1: Figure S1). Overall, the *PNPLA3* p.I148M variant was associated with a significantly increased risk of developing MASLD in the FLAG cohort (common OR = 2.46, *P* = 6.238e‐09). The results of further genotype‐phenotype association tests (all significant with *p* < 0.001) in the FLAG cohort are presented in Table 2. When analysing separately patients recruited in the office‐based practices (*n* = 348) and in university outpatient clinics (*n* = 128) participating in the FLAG program, we detected a high frequency of the *PNPLA3 p.*I148M minor allele in both cohorts: 60.6% and 61.7% of recruited patients carried at least one copy of the [M] allele, respectively. Both groups of patients departed for this reason significantly (both *p* < 0.001) from HWE. Variant *PNPLA3* was associated with an increased risk of developing MASLD in patients recruited in office‐based practices and in the university centres (common OR = 2.42, *P* = 4.294e‐08 and common OR = 2.63, *P* = 4.990e‐07, respectively).

**TABLE 2:** Association tests presenting the risk of developing MASLD in relation to the *PNPLA3* p.I148M polymorphism.

OR statistics	OR (95% CI)	*p* value
[M] ↔ [I]	2.44 (1.84–3.24)	3.009e‐10
[MM] ↔ [II]	7.26 (3.40–15.51)	8.502e‐09
[MM] ↔ [II + IM]	5.80 (2.76–12.18)	2.517e‐07
[MM + IM] ↔ [II]	2.31 (1.62–3.30)	2.610e‐06

*Note:* [M] represents the risk allele for steatosis.

Abbreviations: CI, confidence interval; I, isoleucine; M, methionine; MASLD, Metabolic dysfunction‐associated steatotic liver disease; OR, odds ratio; p, protein (amino acid number); *PNPLA3*, adiponutrin.

### 3.3 *PNPLA3* Genotypes Are Associated With Liver Enzyme Activities and Metabolic Traits

Presence of the *PNPLA3* risk variant was associated with increased ALT (ANOVA *p* = 0.006, Figure 1A) and AST enzyme activities (ANOVA *p* = 0.021, Figure 1B), but not ALP, GGT enzyme activities, or bilirubin levels (all *p* > 0.05). We detected a correlation between the *PNPLA3* p.I148M variant and lower serum triglyceride concentration (ANOVA *p* = 0.003, Figure 2). On the other hand, it did not affect other metabolic traits like serum total cholesterol, LDL, HDL, glucose, HbA1c, or ferritin (all *p* > 0.05).

**FIGURE 1:**
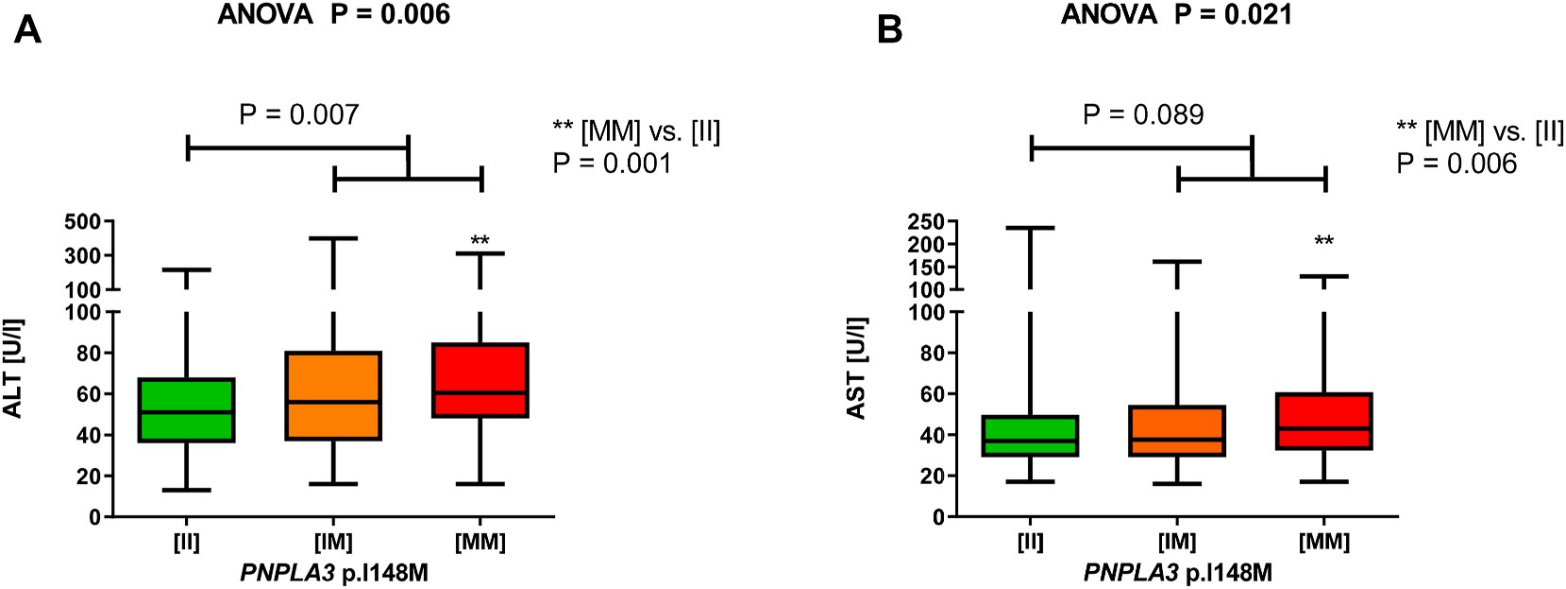
Serum ALT (A) and AST (B) according to the *PNPLA3* genotype.

**FIGURE 2:**
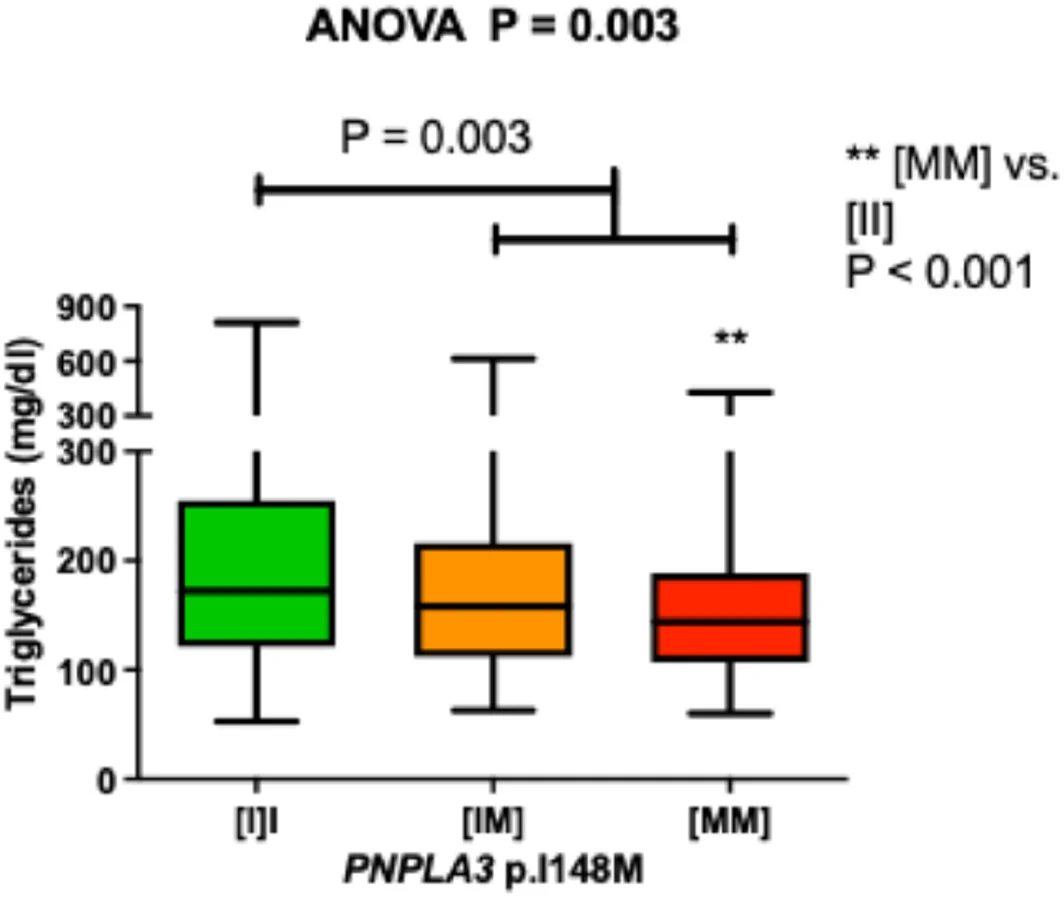
Serum triglyceride concentrations according to *PNPLA3* genotype.

### 3.4 *PNPLA3* Genotypes Are Associated With Non‐Invasive Fibrosis Surrogates

Carriers of the *PNPLA3* p.148MM genotype were more likely to present with LSM values indicating relevant liver fibrosis (i.e., LSM ≥ 9.1 kPa) (OR = 1.98, 95%CI 1.03–3.79, *p* = 0.040). This risk was also increased in patients with diabetes mellitus (OR = 5.45, 95%CI 2.93–10.12, *p* < 0.01) and correlated with BMI (OR = 1.09, 95%CI 1.03–1.15, *p* < 0.01). In multivariate regression model including *PNPLA3* genotype, BMI and diabetes mellitus, all three remained independently associated with LSM ≥ 9.1 kPa: *PNPLA3* p.148MM (OR 2.57, 95%CI 1.22–5.39, *p* = 0.01), BMI (OR 1.07, 95%CI 1.01–1.14, *p* = 0.02), and diabetes mellitus (OR = 4.95, 95%CI 2.55–9.61, *p* < 0.01). A trend was observed for the association between *PNPLA3* p.I148M and FIB‐4 > 2.67 (OR 1.39, *p* = 0.093). No significant association was detected with LSM ≥ 13.0 kPa; however, this result might be related to a relatively low number of patients with LSM above this cut‐off (Table 1).

### 3.5 *HSD17B13* Genotypes Have Only Minor Effect on MASLD Phenotypes

In contrast to *PNPLA3* p.I148M, the *HSD17B13* polymorphism was within the Hardy–Weinberg Equilibrium (*p* > 0.05 according to the exact test, Supporting Information S1: Figure S1). We did not see any difference (*p* > 0.05) in the distribution of the *HSD17B13* genotypes between patients recruited in the office‐based practices and in the university centres (rare variant was detected in 39.0% and 41.4% of individuals, respectively). Among carriers of the *HSD17B13* polymorphism, we detected a trend toward lower ALT (*p* = 0.074, Figure 3A) and significantly lower AST (ANOVA *p* = 0.045, Figure 3B). Serum GGT or ALP enzyme activities were not (both *p* > 0.05) modulated by the *HSD17B13* genotypes. Heterozygotes for this polymorphism had lower FIB‐4 values (*p* = 0.028, Supporting Information S1: Figure S2A) and lower LSM (*p* = 0.034, Supporting Information S1: Figure S2B), but we did not detect any effects on either the lower FIB‐4 cut‐off < 1.30 (*p* = 0.43) or the higher FIB‐4 cut‐off > 2.67 (*p* = 0.094) or on the risk of developing relevant liver fibrosis (i.e., TE ≥ 9.1 kPa) or TE ≥ 13.0 kPa (both *p* > 0.05).

**FIGURE 3:**
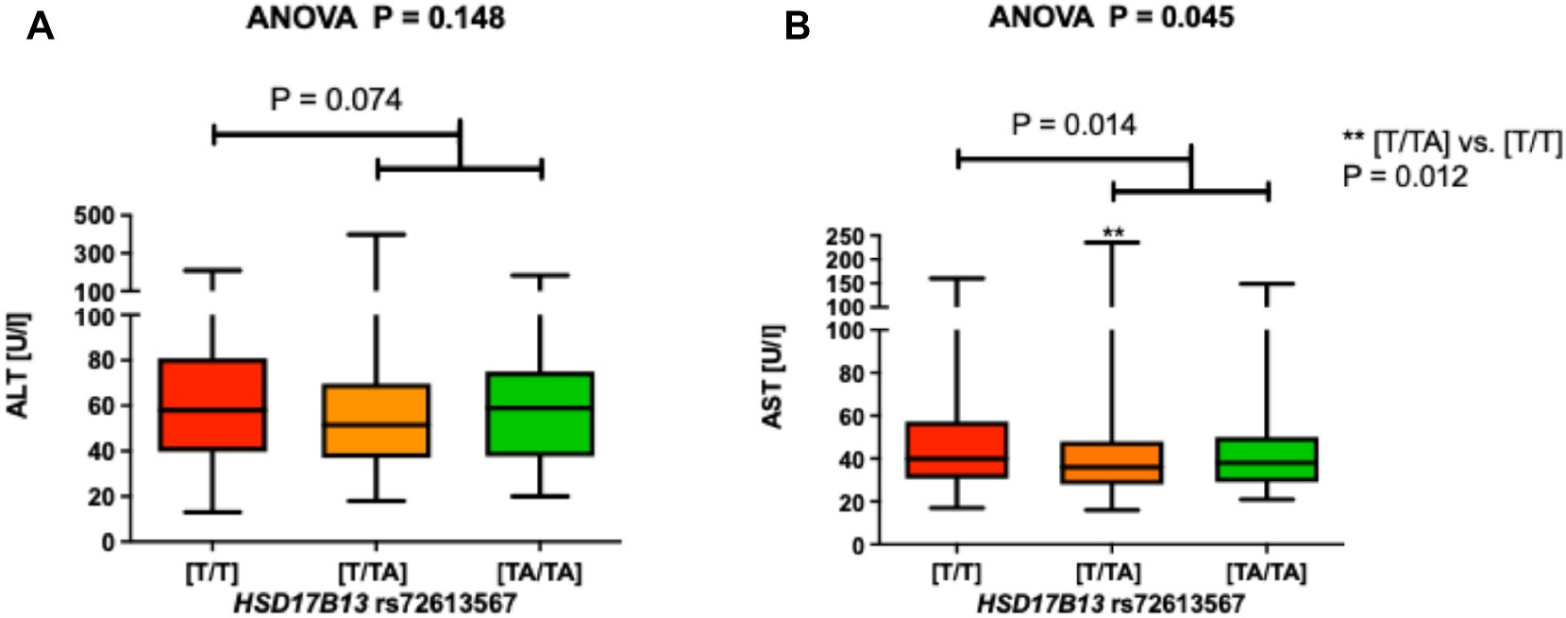
Serum ALT (A) and AST (B) among carriers of different *HSD17B13* genotypes.

### 3.6 Combined Effects of the *PNPLA3* and *HSD17B13* Variants on MASLD Phenotypes in the FLAG Cohort

In the analysis restricted to patients carrying the *PNPLA3* p.148M allele (*n* = 290), we observed that the *HSD17B13* was associated with lower ALT (Figure 4A, ANOVA *p* = 0.023) and AST (Figure 4B, ANOVA *p* = 0.041) but it did not affect the results of LSM or FIB‐4 (both *p* > 0.05). Significantly lower liver enzyme activities were present in particular in *HSD17B13* heterozygotes (Figure 4). In dedicated analyses performed separately in patients recruited at the university centres and in office‐based practices, the *HSD17B13* protective variant was associated with lower serum ALT (*p* = 0.036) and AST (*p* = 0.030) in carriers of the *PNPLA3* minor allele in the latter group only.

**FIGURE 4:**
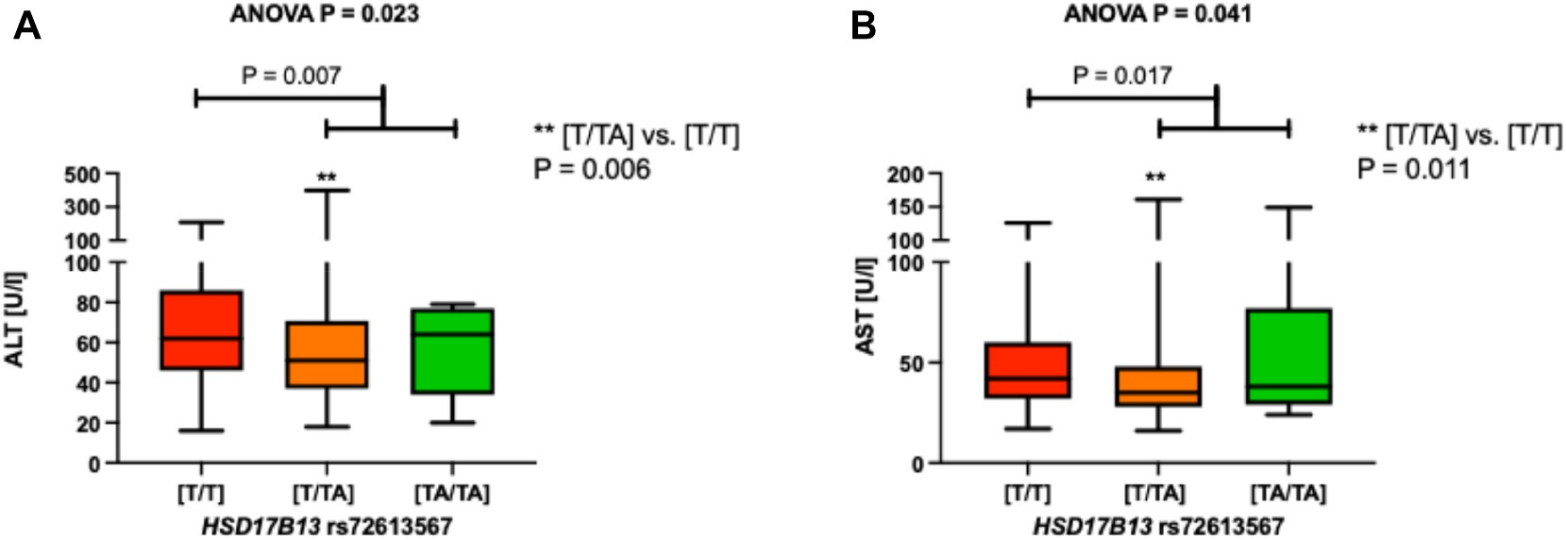
*HSD17B13* genotype is associated with lower ALT (A) and AST (B) among carriers of the *PNPLA3* p.148M allele.

Finally, we computed both variants in a multivariate model. The association between the *PNPLA3* p.I148M polymorphism and liver fibrosis remained significant after adjustment for *HSD17B13*, diabetes and BMI (*p* = 0.013), showing that the *HSD17B13* variant did not fully abrogate the effects of the *PNPLA3* polymorphism.

## 4 Discussion

MASLD is a common liver disease. The development of metabolic liver disease and progression to MASH with significant fibrosis and cirrhosis depend on endogenous and external factors including lifestyle, comorbidities, and inherited predisposition [9, 26]. In the current analysis we analysed the *PNPLA3* and *HSD17B13* variants in patients participating in the German multicenter real‐world MASLD cohort (FLAG study) that was recruited predominantly in secondary care within the German healthcare system (Facharztpraxen). The original FLAG cohort consisted of 507 patients; here, we were able to genotype 476 individuals of whom 348 were recruited in secondary care (office‐based) and 128 in tertiary care (academic outpatient clinics), respectively.

*PNPLA3* and *HSD17B13* genotyping in our MASLD cohort demonstrated at least two major findings. First of all, genetic predisposition affects the path of the patient already before entering the tertiary healthcare level. Secondly, we show that the effects of the *PNPLA3* polymorphism can be somewhat attenuated by the *HSD17B13* variant; however, the major effects of adiponutrin on the development and progression of fatty liver are not abolished by the protective allele.

*PNPLA3* is expressed on hepatic lipid droplets, where it is thought to act as an enzyme that influences the metabolism of stored lipids and promotes lipid accumulation among the carriers of the p.I148M variant [27]. Findings from the last decade, which have been summarised in the recent reviews [28, 29, 30], underscore the role of *PNPLA3* p.I148M as a major genetic trigger and modulator of MASLD. Analysis of the general population included in the UK Biobank showed that this polymorphism might explain 10.8% of the population attributable fraction (PAF) of cirrhosis [31]. In contrast to other studies, which were mostly performed in the tertiary referral units, here we investigated a cohort of MASLD patients that were mostly recruited in secondary care. Of note, already at this level of referral, we detected a very high percentage of patients carrying the *PNPLA3* variant. These individuals were not only at increased risk of developing fatty liver but also had increased markers of liver injury. The effects of *PNPLA3* p.I148M on healthcare utilization were analysed in participants of the Study of Health in Pomerania (SHIP) [32]. Köpp et al. [32]showed that carriers of *PNPLA3* p.I148M had an increased risk of hospitalisation (OR 1.51, 95% CI 1.02–2.15), whereas the second and less frequent polymorphism *TM6SF2* was also linked to the higher outpatient utilisation and inpatient days. This is also in line with the results presented by Arslanow et al. [33]who detected an increased frequency of the *PNPLA3* minor allele among patients referred to a university liver center for detailed workup due to increased liver function tests. Hence, it seems plausible to state that genetic variants involved in the pathogenesis of MASLD modulate the patient's pathway early on through the healthcare system. Indeed, more than 60% of individuals recruited in the FLAG cohort carried at least one copy of the *PNPLA3* polymorphism, which eventually led to a departure of this variant from the Hardy–Weinberg equilibrium.

*HSD17B13* is the second variant which we analysed in our study. It was first detected by Abul‐Husn et al., who showed its potentially protective effects in patients with chronic liver diseases [16]. Notably, this variant was not associated with simple steatosis in this study but reduced the risk of steatohepatitis in MASLD patients. A subsequent histopathological study showed that the *HSD17B13* polymorphism might protect against histological hallmarks of chronic liver injury such as lobular inflammation, ballooning degeneration, and fibrosis [34]. Raja et al. [35]analysed this variant in 587 individuals with chronic liver diseases and demonstrated that it might reduce the risk of developing liver diseases by as much as 50%. A recent biopsy‐based analysis of 227 children and 444 adults showed that it can attenuate the progression of MASLD to MASH [36]. HSD17B13 is involved, among other functions, in the metabolism of retinol and the regulation of sex steroid hormones [17]. In our study, we observed some hepatoprotective effects of *HSD17B13* in MASLD—namely lower AST, and minor effects for markers of liver fibrosis. Of note, the protective effects of *HSD17B13* were especially pronounced among heterozygotes for this variant. *HSD17B13* lowered the harmful effects of the *PNPLA3* polymorphism, but could not fully abrogate the risk of relevant fibrosis conferred by *PNPLA3* in our multivariate analysis. On one hand, we confirmed the protective effect on liver injury as reflected by clinical chemistry markers. Our data are partly in contrast to the protective effects of the *HSD17B13* variant on advanced liver disease and cirrhosis [37]. Overall, *PNPLA3* appears to be the central genetic modifier of the MASLD phenotype. Indeed, recent studies show that *PNPLA3*‐modulation might yield beneficial effects on the fatty liver [38, 39]. While two recently published phase III trials open new avenues for treating patients with MASLD [40, 41], the available data from the post hoc analysis of the MAESTRO‐NASH study [42] do not show major effects of either *PNPLA3* or *HSD17B13* genotypes on the therapeutic outcomes. We acknowledge the limitations of our study. First, larger cohorts may be required to capture the effects of both variants more precisely, particularly *HSD17B13*. The same applies to analyses of liver biopsy specimens, which were not available in our patients but could allow more precise phenotyping characterisation of the cohort. Finally, inclusion of patients with hepatic decompensation or HCC would provide a more real‐world insight into the clinical impact of the studied variants in an office‐based setting. Additionally, the choice of non‐invasive technologies to assess the fibrosis stage and risk classify patients introduces variations inherent to non‐invasive testing in this real‐world cohort [43].

Based on the results of our study, we recommend that patients with the minor *PNPLA3* p.I148M allele should receive special attention since they are at risk of developing more aggressive liver disease, which can be detected already at the level of the office‐based practices. In particular, homozygotes for the minor *PNPLA3* allele are prone to increased liver injury. These effects seem to be only slightly modulated by protective variants, such as *HSD17B13*. Knowledge of the patient's *PNPLA3* genotype might, at least to a certain extent, guide their path through the healthcare system. Hence, *PNPLA3* p.I148M can guide individual decision making and the diagnostic approach to patients with MASLD.

In conclusion, the *PNPLA3* p.I148M polymorphism is associated with a more pronounced liver phenotype in patients seen in secondary care. These effects might be slightly lower in carriers of the *HSD17B13* polymorphism. Genetic testing might help to detect patients carrying the inherited predisposition earlier and allow for intensified management.

## Conflicts of Interest

The authors declare no conflicts of interest.

## Supporting information


Supporting Information S1

## Data Availability

The data that support the findings of this study are available on request from the corresponding author. The data are not publicly available due to privacy or ethical restrictions.
