# Supplementary material for: Metabolic Dysfunction‐Associated Steatotic Disease and Genetic Risk in Germany—An Observational Real‐World Cohort Study
Source: United European Gastroenterol J. 2026 Oct 7;14(8):e70298. doi: 10.1002/ueg2.70298 (PMC13643938; doi:10.1002/ueg2.70298)
Supplement: Supplementary file 1 [file UEG2-14-e70298-s001.docx]

**Supplementary Figure 1**

De Finetti diagram with Hardy-Weinberg parabola for the *PNPLA3* (rs738409, black line) and *HSD17B13* (rs72613567, blue line) polymorphisms.

*De Finetti diagram with Hardy-Weinberg parabola for both variants in the FLAG cohort. Frequencies of the minor alleles are shown at the intersection between the vertical dotted line and the bottom perpendicular. The frequencies of major and minor allele homozygotes are depicted on the left and right diagonal axes, respectively, whereas the frequency of heterozygotes for each variant are plotted on the vertical axis on the left. Genotype frequencies of HSD17B13 plot on the diagram’s parabola, i.e. they are in the Hardy-Weinberg Equilibrium (HWE, P > 0.05). Genotype frequencies of PNPLA3 plot, in turn, outside the parabola and deviate significantly (P < 0.001) from HWE. This is due to the overrepresentation of the PNPLA3 allele [M] which is associated with the increased risk of developing fatty liver. Plot drawn using online available tool (http://ihg.gsf.de/cgi-bin/hw/hwa1.pl).*

**Supplementary Figure 2**

FIB-4 (A) and liver stiffness by transient elastography (B) according to *HSD17B13* genotype.

**Supplementary Figure 2A**

**Supplementary Figure 2B**
